# Supplementary material for: Peripherally Restricted Activation of Opioid Receptors Influences Anxiety-Related Behaviour and Alters Brain Gene Expression in a Sex-Specific Manner
Source: Int J Mol Sci. 2024 Dec 7;25(23):13183. doi: 10.3390/ijms252313183 (PMC11642130; doi:10.3390/ijms252313183)
Supplement: Supplementary file 1 [file ijms-25-13183-s001.zip › ijms-3311485-supplementary.pdf]

Table S1. *Differentially expressed genes in the proximal colon of loperamide vs control group based on significance (FDR < 0.05 and |logFC| > 0.585).*

Gene	logFC	FDR	p value
Apol9a	-1.0921	1.34E-13	1.36E-17
Fam118a	0.65062	1.02E-12	4.12E-16
RGD1565059	0.64664	3.67E-12	1.85E-15
Maff	0.99014	1.05E-11	6.37E-15
Sgk1	-1.5693	1.22E-10	9.85E-14
Fitm2	-0.7509	1.24E-10	1.19E-13
Il7	0.72686	1.24E-10	1.25E-13
Plekhg6	-1.0212	1.02E-09	1.65E-12
Tsku	-0.7372	1.93E-09	3.97E-12
Fgf19	-5.5678	1.93E-09	4.10E-12
Ing3	0.62332	2.96E-09	6.56E-12
Mthfs	0.5904	4.72E-09	1.10E-11
	-1.0426	6.57E-09	1.79E-11
Itga5	-0.8361	7.45E-09	2.10E-11
Dnajb3	-0.8216	9.11E-09	2.67E-11
LOC108348086	-0.9134	1.70E-08	5.31E-11
Kcnf1	-2.1639	2.08E-08	6.91E-11
Hsd3b3	-1.0214	4.11E-08	1.54E-10
Cyp26a1	-1.6664	5.48E-08	2.10E-10
Fam169b	1.2429	6.06E-08	2.39E-10
Dhrs9	0.65092	1.39E-07	6.46E-10
	1.21511	1.89E-07	9.17E-10
Wnt11	-0.7504	1.99E-07	1.01E-09
Gpr4	-0.8825	3.11E-07	1.63E-09
Nr0b2	-1.6427	4.04E-07	2.24E-09
Gvin1	0.7603	5.26E-07	3.13E-09
Tlr4	0.65056	7.63E-07	4.85E-09
Kansl1l	0.71651	8.26E-07	5.42E-09
Mat2a	-0.6775	8.58E-07	5.71E-09
Mab21l4	0.67404	1.24E-06	9.11E-09
Mmp10	0.67516	2.40E-06	2.08E-08
C2cd4b	-1.7193	2.49E-06	2.21E-08
Gja5	-1.6698	2.86E-06	2.82E-08
Cyp8b1	1.10978	5.16E-06	6.04E-08
Ankrd34a	-1.1294	8.68E-06	1.15E-07
Rhod	-0.9035	1.13E-05	1.56E-07
Ifit1bl	0.70668	1.85E-05	2.74E-07
B4galt1	-0.9901	2.19E-05	3.38E-07
Fut9	0.66358	3.78E-05	6.95E-07

	1.64469	4.62E-05	8.60E-07
Sln	-1.1066	4.62E-05	8.66E-07
LOC103690878	-0.9361	4.74E-05	8.99E-07
Bsnd	-2.9747	5.45E-05	1.08E-06
RGD1308544	0.66294	6.66E-05	1.36E-06
Celf3	-0.647	6.96E-05	1.48E-06
Aqp8	-3.0236	7.40E-05	1.57E-06
Pygm	-0.6087	8.60E-05	1.91E-06
Nos3	-0.6149	9.64E-05	2.25E-06
Prap1	-1.4548	0.00012	2.95E-06
Gp2	-1.6895	0.00013	3.33E-06
Exoc3l2	-0.6723	0.00015	3.79E-06
Tac1	-0.6638	0.00016	4.12E-06
Nell1	-0.7953	0.00016	4.39E-06
Tacstd2	0.65415	0.00017	4.49E-06
Emp3	-0.6096	0.00019	5.29E-06
Lgi1	-0.7419	0.00019	5.44E-06
Adra1d	-0.8609	0.0002	5.90E-06
Lrrc4	-1.2941	0.00029	9.32E-06
Vxn	-0.8572	0.00031	1.02E-05
	0.67903	0.00032	1.06E-05
Armcx2	-0.6089	0.00033	1.10E-05
Ifit3	0.75419	0.00034	1.13E-05
Efcc1	-0.7643	0.00036	1.24E-05
Atp2b2	-0.7699	0.00038	1.32E-05
	0.74311	0.00041	1.44E-05
Clec2e	0.61835	0.00047	1.68E-05
Numbl	-0.6048	0.00047	1.68E-05
Adra2a	-0.5887	0.00056	2.05E-05
Slc37a2	-1.7784	0.00057	2.11E-05
Gpr150	0.63852	0.00057	2.11E-05
	2.86275	0.00057	2.15E-05
Cyp4f5	5.32243	0.00059	2.22E-05
Slc16a14	-0.8639	0.0006	2.28E-05
Slc6a14	0.79729	0.00067	2.62E-05
Duoxa2	-1.2098	0.00072	2.84E-05
Baiap2l2	-2.197	0.00076	3.07E-05
C2cd4a	-1.138	0.00078	3.19E-05
Slc5a12	2.24942	0.00081	3.32E-05
Tceanc	0.59328	0.00081	3.35E-05
Cyp2d4	-0.6424	0.00083	3.44E-05
Macc1	0.78152	0.00088	3.72E-05

Tubb4a	-0.6232	0.00089	3.75E-05
Cyp2b1	-1.6582	0.00101	4.36E-05
Per3	0.73489	0.00104	4.58E-05
Rasl12	-0.6027	0.00104	4.60E-05
Erich5	1.74307	0.0011	4.89E-05
Foxs1	-0.9113	0.0011	4.93E-05
Gbp3	0.96989	0.00111	4.99E-05
Lrrc69	0.68302	0.00135	6.39E-05
Ceacam1	-0.8697	0.00148	7.22E-05
RGD1561916	-0.7329	0.00159	7.96E-05
Akap3	-0.9027	0.00169	8.59E-05
Hoxc6	0.89455	0.00174	8.91E-05
Dbp	0.99469	0.00186	9.72E-05
Acsbg2	0.79144	0.00197	0.00011
Samd5	-1.4397	0.00197	0.00011
Peg12	-0.8263	0.00206	0.00011
Tex11	1.07689	0.00217	0.00012
LOC103690302	-0.6659	0.00226	0.00013
LOC100910656	-1.0916	0.00229	0.00013
Rfx8	0.77558	0.00238	0.00014
Nr4a1	-1.2364	0.00257	0.00015
Amn	-0.9903	0.00268	0.00016
Tmem121	-0.876	0.0027	0.00016
Il1a	0.81959	0.00275	0.00017
Sult1c3	-2.532	0.00287	0.00018
Fosb	-1.9617	0.00326	0.00021
LOC689065	0.60324	0.00345	0.00023
Chst8	-0.8335	0.00361	0.00024
Lrrn1	-0.6627	0.00364	0.00024
Cox6a2	-0.8127	0.00371	0.00025
Rbp7	-0.8837	0.00403	0.00028
Pls3	-0.7709	0.00415	0.00029
S100a9	0.83713	0.00426	0.0003
Bmp8a	-0.8206	0.00441	0.00032
Asb15	-1.9469	0.00463	0.00034
Exd1	0.71641	0.00474	0.00035
	1.11865	0.00487	0.00036
Acot6	0.79455	0.00494	0.00037
Lgsn	1.02215	0.00533	0.00041
Six2	-2.0246	0.00546	0.00043
Cd180	0.83754	0.00558	0.00044
	-0.8209	0.00588	0.00047

Ms4a4c	0.96136	0.0063	0.00052
Tef	0.59777	0.00636	0.00053
Agtr2	-1.2545	0.00643	0.00053
Elf5	-0.8434	0.00659	0.00055
	-0.6828	0.0071	0.00061
Lypd6	-0.5923	0.00757	0.00067
	0.60967	0.0081	0.00074
Rbp2	-0.8138	0.00821	0.00076
Sgk2	-0.7619	0.00848	0.00079
Has1	-1.0117	0.00868	0.00082
Ntsr1	-0.6154	0.00928	0.0009
	1.16779	0.00948	0.00093
	0.86294	0.00949	0.00093
	0.92237	0.0102	0.00102
Ciart	1.14743	0.01025	0.00102
	2.16517	0.01162	0.00121
Sp5	1.158	0.01222	0.00129
Pou2f3	-0.9928	0.0127	0.00135
B3gnt7	-1.3582	0.01277	0.00136
Naaladl1	-1.104	0.01327	0.00143
	1.47935	0.01327	0.00143
Hmx3	-0.8032	0.01398	0.00153
Lhfpl4	-1.1215	0.01411	0.00155
	1.79987	0.01422	0.00158
	-0.6429	0.01426	0.00159
Camp	-0.6908	0.01426	0.00159
Apoa1	-0.9773	0.01478	0.00167
	0.63795	0.01553	0.00177
	0.95103	0.01615	0.00188
	2.07856	0.01678	0.00197
Snca	-0.6632	0.01697	0.002
Sec1	-2.0988	0.01772	0.00214
Lama1	0.60969	0.01854	0.00226
Pdzd3	-0.7522	0.01872	0.0023
Bmp15	-0.5971	0.01877	0.00231
Lhx8	-1.3227	0.01929	0.00239
Mttp	-1.5293	0.01967	0.00244
Kcns1	0.66585	0.01983	0.00247
	-0.7283	0.01983	0.00248
Tnnc2	-0.8236	0.01996	0.0025
Gpr174	0.60108	0.01998	0.00251
Gal3st2	-2.118	0.02072	0.00266

	1.1639	0.02146	0.0028
Slc2a5	0.79905	0.02199	0.00291
Lrp2	0.58663	0.02205	0.00292
Bnc1	1.0542	0.02271	0.00305
	0.69639	0.02315	0.00311
	-2.0314	0.02329	0.00316
	0.94357	0.0235	0.0032
	1.09064	0.02389	0.00328
Cd207	0.81179	0.02423	0.00336
Tmem151b	-0.7412	0.02431	0.00338
Fgf22	-0.7537	0.02473	0.00346
RGD1561102	0.82478	0.02493	0.0035
	-1.5938	0.02519	0.00355
Plg	0.77055	0.02585	0.00369
Mal	-1.1841	0.02596	0.00372
Krt77	1.02668	0.02612	0.00376
	1.12189	0.02648	0.00384
Gdf6	0.74309	0.02653	0.00385
Pth	0.99924	0.02704	0.00396
Grik3	-0.6494	0.02704	0.00397
Epor	-0.7371	0.02704	0.00397
Cyp1a1	-0.7792	0.02803	0.00415
Ngb	-0.9619	0.02829	0.0042
	1.6083	0.02845	0.00424
Epm2a	0.67081	0.02846	0.00424
Cyp24a1	-1.5633	0.02855	0.00427
Klrb1c	0.60546	0.02994	0.00458
Sh2d7	-0.9761	0.03032	0.00467
Fam71f2	0.9789	0.03049	0.0047
	1.35333	0.03086	0.00477
Nts	-1.5612	0.03095	0.00479
	-0.8123	0.03133	0.00489
Mmp12	0.59762	0.03216	0.00508
	-1.7741	0.0331	0.00528
	1.06176	0.03355	0.00539
Spink4	-1.4126	0.03361	0.00541
Diras1	-0.8762	0.03385	0.00546
Lcn5	-2.5022	0.03385	0.00547
Klrb1	0.86338	0.03416	0.00556
Pla2g2d	-0.6041	0.0344	0.0056
Upk3a	0.85334	0.03447	0.00563
Krt80	-0.6556	0.03468	0.00567

	-1.8855	0.03516	0.0058
Iglon5	-0.5954	0.03532	0.00584
Fam181b	-0.7868	0.03723	0.00628
Slc7a11	0.60112	0.03754	0.00636
Igf2bp3	-0.9171	0.03828	0.00655
Crb2	0.80369	0.03916	0.00674
	-0.6422	0.03977	0.00686
	-1.2368	0.04001	0.00692
	1.91619	0.04144	0.00733
	1.48344	0.04154	0.00736
Acot1	-0.606	0.04173	0.0074
Gnat3	-1.2377	0.04209	0.00748
Isl1	0.67512	0.04233	0.00755
Tmprss12	0.64979	0.04272	0.00763
Cxcr3	0.59314	0.0429	0.00768
Ascl2	-1.0104	0.04472	0.0081
Oprl1	-0.6104	0.0452	0.0082
Slit1	-0.6777	0.04628	0.00852
Fabp2	-0.692	0.04637	0.00854
Slc7a14	-0.5945	0.04815	0.009
Amy1a	-1.29	0.04887	0.00918
Tpbgl	-0.588	0.04932	0.0093

Table S2. *a) Top 40 differentially expressed genes in the proximal colon of male rats (FDR < 0.05 and |logFC| > 0.585). n=8 rats per treatment group.*

Gene	logFC	FDR	PValue	Expression
**RGD1565059**	0.809159363	5.28E-09	5.32E-13	Increased in Loperamide
**Fam118a**	0.690889485	7.58E-07	3.06E-10	Increased in Loperamide
**Apol9a**	-1.093673469	8.08E-07	4.08E-10	Decreased in Loperamide
**Bcl9l**	-0.589863545	1.17E-06	8.27E-10	Decreased in Loperamide
**Fitm2**	-0.852574492	1.17E-06	7.70E-10	Decreased in Loperamide
**Ppp2r5b**	-0.682169222	1.23E-06	9.91E-10	Decreased in Loperamide

**Plekhg6**	-1.208234377	1.31E-06	1.19E-09	Decreased in Loperamide
**Ing3**	0.742990975	2.58E-06	2.61E-09	Increased in Loperamide
**Maff**	1.024391959	3.58E-06	3.97E-09	Increased in Loperamide
**Mthfs**	0.693058674	5.22E-06	6.32E-09	Increased in Loperamide
**Arrdc3**	0.597170866	1.65E-05	2.50E-08	Increased in Loperamide
**Plin3**	-0.590688557	2.33E-05	4.22E-08	Decreased in Loperamide
**Tsku**	-0.790842687	2.72E-05	5.50E-08	Decreased in Loperamide
**Il7**	0.725549886	3.52E-05	7.45E-08	Increased in Loperamide
**Tlr4**	0.804038911	5.17E-05	1.38E-07	Increased in Loperamide
**Kcnf1**	-2.397073047	5.17E-05	1.34E-07	Decreased in Loperamide
**Fgf19**	-6.139562686	5.17E-05	1.45E-07	Decreased in Loperamide
**Plekhg2**	-0.618643848	6.46E-05	2.41E-07	Decreased in Loperamide
**Bcar3**	0.6752945	6.83E-05	2.96E-07	Increased in Loperamide
**Nudt22**	-0.618959778	6.83E-05	2.67E-07	Decreased in Loperamide
**Bcorl1**	-0.674063619	6.83E-05	2.93E-07	Decreased in Loperamide
**Mat2a**	-0.819385067	6.83E-05	2.84E-07	Decreased in Loperamide
**Hsd3b3**	-1.120783613	6.83E-05	2.83E-07	Decreased in Loperamide
**Bcl2l11**	0.62687217	7.74E-05	3.75E-07	Increased in Loperamide
**LOC108348086**	-0.95159035	0.000113611	5.96E-07	Decreased in Loperamide
**Gpr160**	0.698650908	0.000123501	6.60E-07	Increased in Loperamide
**Klf11**	0.630509997	0.000132053	7.75E-07	Increased in Loperamide

**Ankrd34a**	-1.399574137	0.000263074	1.75E-06	Decreased in Loperamide
**Gvin1**	0.835861198	0.000282593	2.02E-06	Increased in Loperamide
**Itga5**	-0.801576194	0.000337613	2.62E-06	Decreased in Loperamide
**Fdxacb1**	0.615982598	0.000406655	3.32E-06	Increased in Loperamide
**Wnt11**	-0.77579504	0.000446391	3.78E-06	Decreased in Loperamide
**Dhrs9**	0.661211214	0.000513641	4.60E-06	Increased in Loperamide
**Cyp26a1**	-1.627249281	0.000513641	4.61E-06	Decreased in Loperamide
**Cyp8b1**	1.28156516	0.000589248	5.76E-06	Increased in Loperamide
**Gpr4**	-0.886586827	0.000589248	5.77E-06	Decreased in Loperamide
**Tceanc**	0.884451202	0.00085146	9.11E-06	Increased in Loperamide
**Fam169b**	1.174567611	0.000879755	9.59E-06	Increased in Loperamide
**Alas1**	-0.590557937	0.000922436	1.02E-05	Decreased in Loperamide
**Scd2**	-0.659805939	0.000922665	1.04E-05	Decreased in Loperamide

b) Top 40 differentially expressed genes in the proximal colon of female rats (FDR < 0.05 and |logFC| > 0.585). n=7-8 rats per treatment group.

**Gene**	**logFC**	**FDR**	**PValue**	**Expression**
**Sgk1**	-1.943497391	3.46E-06	4.83E-10	Decreased in Loperamide
**Dnajb3**	-1.121197457	3.46E-06	6.97E-10	Decreased in Loperamide
**Apol9a**	-1.090214877	2.00E-05	6.04E-09	Decreased in Loperamide
**Fam118a**	0.609000077	0.000369411	1.86E-07	Increased in Loperamide
**Maff**	0.95230172	0.000443141	2.94E-07	Increased in Loperamide
**Il7**	0.728824099	0.000443141	3.42E-07	Increased in Loperamide

**AABR07002677.2**	-1.176075439	0.000443141	3.82E-07	Decreased in Loperamide
**Itga5**	-0.886794726	0.000830182	1.30E-06	Decreased in Loperamide
**Celf3**	-0.958745089	0.000830182	1.35E-06	Decreased in Loperamide
**Nceh1**	-0.67817898	0.001165931	2.24E-06	Decreased in Loperamide
**Fgf19**	-5.290924354	0.001233225	2.65E-06	Decreased in Loperamide
**Epha2**	0.656988671	0.001923533	4.66E-06	Increased in Loperamide
**Fam169b**	1.332752607	0.001984979	5.01E-06	Increased in Loperamide
**Cyp26a1**	-1.717416087	0.003290278	1.03E-05	Decreased in Loperamide
**Tsku**	-0.665893413	0.004602289	1.85E-05	Decreased in Loperamide
**Gp2**	-2.322349114	0.004602289	2.00E-05	Decreased in Loperamide
**Fitm2**	-0.629353529	0.004905769	2.18E-05	Decreased in Loperamide
**Lrrc32**	-0.735788632	0.004946857	2.25E-05	Decreased in Loperamide
**Zhx2**	-0.728298959	0.004964717	2.35E-05	Decreased in Loperamide
**Nr0b2**	-1.696588812	0.005045521	2.44E-05	Decreased in Loperamide
**LOC108348086**	-0.857892362	0.005157375	2.55E-05	Decreased in Loperamide
**Dhrs9**	0.640418674	0.006229083	3.27E-05	Increased in Loperamide
**Slc6a20**	0.665261263	0.007561634	4.45E-05	Increased in Loperamide
**Nhsl2**	-0.749900449	0.008584005	5.46E-05	Decreased in Loperamide
**Wnt11**	-0.7205387	0.009526039	6.44E-05	Decreased in Loperamide
**Gpr4**	-0.882076358	0.010203728	7.10E-05	Decreased in Loperamide
**Usp2**	-0.677496784	0.012675118	9.46E-05	Decreased in Loperamide
**Mab21l4**	0.665220484	0.012965927	0.000100734	Increased in Loperamide

**Kansl1l**	0.693333544	0.01326845	0.000105762	Increased in Loperamide
**Emp3**	-0.763871641	0.013486473	0.00010977	Decreased in Loperamide
**Hoxc6**	1.295314762	0.013486473	0.000110221	Increased in Loperamide
**C2cd4b**	-1.72434346	0.014613742	0.000129756	Decreased in Loperamide
**Nell1**	-0.959829835	0.016152374	0.000148779	Decreased in Loperamide
**Plekhg6**	-0.79721918	0.016684796	0.000158245	Decreased in Loperamide
**AABR07062068.1**	1.117094849	0.017147452	0.000164364	Increased in Loperamide
**Bsnd**	-3.26849119	0.018346516	0.000194368	Decreased in Loperamide
**Hsd3b3**	-0.869921827	0.018571773	0.000200502	Decreased in Loperamide
**Mmp10**	0.649163583	0.019882556	0.000228697	Increased in Loperamide
**Pygm**	-0.690560007	0.020032766	0.000234467	Decreased in Loperamide
**Pla2g2d**	-1.21235774	0.020228494	0.000243904	Decreased in Loperamide
